# DRUID: a pipeline for transcriptome-wide measurements of mRNA stability

**DOI:** 10.1261/rna.062877.117

**Published:** 2018-05

**Authors:** Andrew Lugowski, Beth Nicholson, Olivia S. Rissland

**Affiliations:** 1 Molecular Medicine Program, The Hospital for Sick Children Research Institute, Toronto, Ontario M5G 0A4, Canada; 2 Department of Molecular Genetics, University of Toronto, Toronto, Ontario M5S 1A8, Canada

**Keywords:** metabolic labeling, mRNA decay, bioinformatics

## Abstract

Control of messenger RNA (mRNA) stability is an important aspect of gene regulation. The gold standard for measuring mRNA stability transcriptome-wide uses metabolic labeling, biochemical isolation of labeled RNA populations, and high-throughput sequencing. However, difficult normalization procedures have inhibited widespread adoption of this approach. Here, we present DRUID (for determination of rates using intron dynamics), a new computational pipeline that is robust, easy to use, and freely available. Our pipeline uses endogenous introns to normalize time course data and yields reproducible half-lives, even with data sets that were otherwise unusable. DRUID can handle data sets from a variety of organisms, spanning yeast to humans, and we even applied it retroactively on published data sets. We anticipate that DRUID will allow broad application of metabolic labeling for studies of transcript stability.

## INTRODUCTION

A critical component in controlling gene expression, RNA decay is essential for nearly all biological processes, from early development to inflammatory responses (Giraldez et al. 2006; Tadros et al. 2007; Brooks and Blackshear 2013). However, transcriptome-wide measurements of mRNA half-lives have long been challenging and represent a major barrier for broad investigations of how mRNA stability is regulated. One strategy has been to shut off transcription, thereby repressing synthesis of all transcripts. In *Saccharomyces cerevisiae*, these experiments typically involve using a temperature-sensitive mutant of RNA polymerase II (Herrick et al. 1990; Grigull et al. 2004; Presnyak et al. 2015), and work in mammalian cell lines has predominantly relied upon drugs that target RNA polymerases, such as actinomycin D and α-amanitin (Ross 1995; Bensaude 2011). Each of these treatments places substantial stress on the cell and can alter the stability and localization of numerous transcripts, as well as broader phenotypes like cell growth (Bensaude 2011; Sun et al. 2013; Geisberg et al. 2014).

**FIGURE 1.**
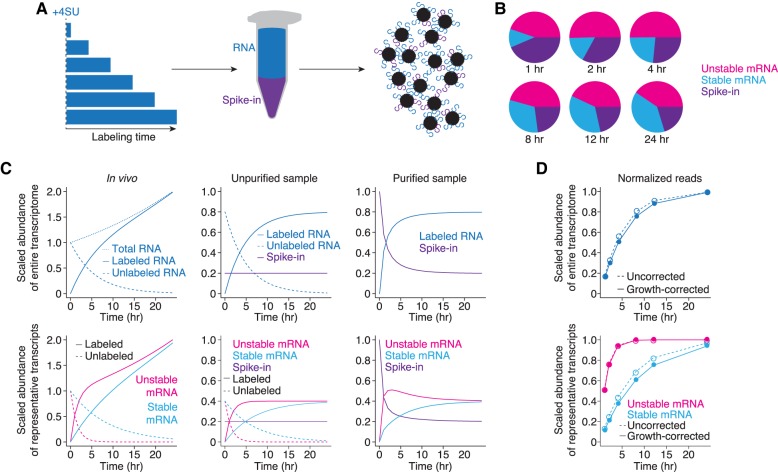
Conceptual underpinnings of the approach-to-equilibrium method. (*A*) Representation of the approach-to-equilibrium experimental method. Cells are incubated with 4SU for increasing amounts of time. Isolated RNA is then biotinylated in vitro and purified using streptavidin. The eluate is then prepared for high-throughput RNA sequencing. (*B*) Representation of typical RNA-seq results. Reads for each gene represent a fraction of the total library, which changes through time, depending on individual synthesis and decay rates as well as overall transcription levels. Representative results are shown for an unstable transcript (pink), a stable transcript (blue), and a spike-in (purple). (*C*) Simulation of abundances during an approach-to-equilibrium experiment for the transcriptome (*top*) and individual transcripts (*bottom*), scaled to the total abundance of the transcriptome (*top*) or the total abundance of an individual transcript (*bottom*) when labeling starts. (*Left*) As cells are incubated longer with 4SU, the labeled RNA population increases at a rate sufficient to replace the unlabeled population and for cell growth. The unlabeled population for unstable transcripts (pink) necessarily decreases faster than for stable transcripts (blue). (*Center*) Once the RNA is extracted, spike-ins are added at a constant ratio to the total amount of RNA, and information about cell growth is lost. (*Right*) After purification, the ratio of spike-in to labeled sample behaves stereotypically with a rapid decrease over the initial time points and convergence to an asymptote, which represents the ratio of the RNAs in the unpurified sample. Note that at early time points, unstable transcripts represent a larger fraction of the library than their eventual steady-state ratios. (*D*) Simulation of normalization strategies. The pie charts (from *B*) are normalized to spike-in levels to calculate the relative abundance of the entire transcriptome or specific transcripts, as appropriate. These measurements can then be growth-corrected, and, using a bounded growth equation, half-lives can be calculated. Note that correcting for growth has a larger effect on stable transcripts than on unstable ones.

Metabolic labeling has emerged as a powerful alternative strategy for determining RNA stabilities under more physiological conditions (Ross 1995; Rabani et al. 2011; Tani et al. 2012; Neymotin et al. 2014; Duffy et al. 2015). This approach uses nucleobase or nucleoside analogs, such as 4-thiouridine (4SU), 5-bromouridine, or 5-ethynyl uridine, all of which allow subsequent isolation of labeled RNA populations (Rabani et al. 2011; Tani et al. 2012; Paulsen et al. 2013; Imamachi et al. 2014; Neymotin et al. 2014; Duffy et al. 2015). The selected RNA is then quantified, often by high-throughput sequencing. Several experimental variations of metabolic-labeling methods have been previously described (Munchel et al. 2011; Tani et al. 2012; Imamachi et al. 2014; Neymotin et al. 2014; Paulsen et al. 2014; Herzog et al. 2017). One method uses an approach-to-equilibrium strategy (Fig. 1A). Here, cells are harvested after increasing times of incubation with the analog. Over the time course, transcript abundance of the labeled population approaches steady-state levels. Because the rate of this approach is determined by transcript stability, these measurements can be used to infer half-lives (Greenberg 1972; Ross 1995; Neymotin et al. 2014).

However, metabolic labeling has been used with mixed success for a number of reasons. First, although approach-to-equilibrium experiments have been successfully used together with microarrays (Friedel et al. 2009; Tippmann et al. 2012; Duan et al. 2013), RNA-seq–based quantitation and analysis is more challenging due to the inherent compositional nature of high-throughput sequencing (see below). Second, metabolic labeling experiments require spike-ins that are not commercially available, but these are essential, especially in the case of quantitation using RNA-seq. Third, the resultant data are complicated and less easily analyzed than shut-off experiments whose results can be fit to simple exponential decay models. Thus, despite the clear benefits of metabolic labeling, RNA polymerase inhibitors remain much more broadly used (Burow et al. 2015; Kumagai et al. 2016; Mauer et al. 2016; Ayupe and Reis 2017).

Here, we describe DRUID, a computational method for determining transcript half-lives on a transcriptome-wide scale using metabolic labeling. By normalizing to intron-mapping reads, our method allowed us to determine mRNA stabilities with higher reproducibility and ease than other methods. Because DRUID makes use of endogenous normalization standards, it can be used with any approach-to-equilibrium labeling and purification approach. Our results also suggest that variation between replicates in metabolic labeling experiments is partly due to technical differences that can be overcome through the use of these internal standards. Underscoring this conclusion, DRUID can also rescue poorly behaving, and previously unusable, data sets, and can even be applied to data sets from species with few introns, such as *S. cerevisiae*. Finally, we have developed a computational package that is publicly available to enable broader use by the community.

## RESULTS

### The conceptual underpinnings of metabolic labeling and approach-to-equilibrium kinetics

The approach-to-equilibrium strategy relies upon incorporation of a nucleotide analog into nascent transcripts so that, with increasing incubation periods, the fraction of transcripts labeled increases until steady state has been reached. The RNA-seq readout of metabolic-labeling experiments can be envisioned as a series of pie charts through time with each slice representing the relative proportion of reads mapping to each transcript (Fig. 1B). Unlike most RNA-seq experiments, where only a handful of transcripts will change in abundance, during metabolic labeling experiments, the relative and absolute abundance of every transcript will change over the experiment (Fig. 1C). For an individual transcript, its relative abundance is determined by synthesis and decay rates, but the absolute abundance of the total labeled population increases with longer exposure to the nucleotide analog at a rate sufficient to replace degraded, unlabeled RNA as well as to allow for cell growth (Fig. 1C, left panels). Determining transcript half-lives requires taking both classes of behaviors into account.

The current solution uses exogenously added spike-ins to convert relative RNA-seq measurements into absolute measurements of RNA abundance (Fig. 1C, middle panels). Typically, spike-ins are added to the harvested RNA from each time point prior to biochemical purification, and information about growth rate is necessarily lost at this step. Because the proportion of spike-ins to the total RNA sample remains constant, once the labeled population has been purified in vitro, the proportion of spike-ins decreases over time while that of the labeled sample increases. These ratios approach that in the original, unpurified sample (Fig. 1C, right panels). Individual transcripts will reach equilibrium with differing kinetics, determined entirely by their stability, with the most unstable transcripts reaching equilibrium the fastest. To calculate half-lives, the RNA-seq libraries are normalized to the spike-ins and further corrected for cell growth. A bounded growth equation is used to infer the decay rate of the unlabeled RNA population (Fig. 1D). Thus, the normalization scheme influences both the magnitude of the calculated half-lives as well as the number of genes for which half-lives can be determined (because poor normalization will give behavior that is not easily fit by a bounded growth equation).

Spike-ins are thus absolutely essential for determining half-lives using metabolic labeling. Although there are commercially available RNA-seq spike-ins (such as the ERCC set), these lack nucleotide analogs, such as 4SU, and so currently each laboratory in vitro transcribes their own standards, as needed (Imamachi et al. 2014; Neymotin et al. 2014). However, there is no broadly accepted standard for these spike-ins, in terms of the number of transcripts, their nucleotide make-up, or length, and variations in spike-in make-up can have large effects on the eventual half-life determination.

### Normalizing to exogenous whole organism spike-ins allows for the calculation of RNA half-lives

We identified two major barriers for the acceptance of the approach-to-equilibrium method: the cost, difficulty, and technical variation associated with current spike-ins; and the computational complexities for analyzing subsequent data sets. Our goal was to develop a method easy enough for widespread use of metabolic labeling, and so we set about reducing these barriers.

**FIGURE 2.**
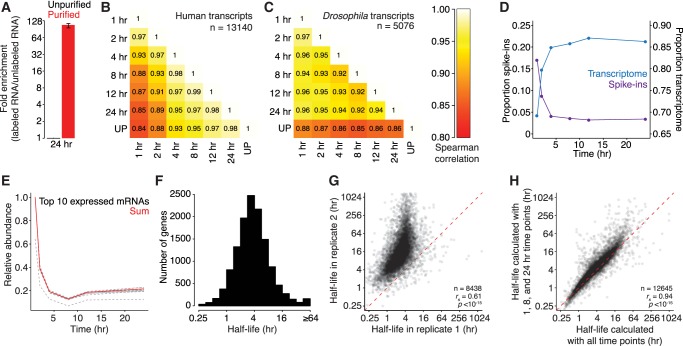
Exogenous, whole-organism spike-ins can be used to calculate transcript half-lives. (*A*) Enrichment of 4SU-labeled transcripts. Plotted is the mean enrichment of *Drosophila* S2 RNA compared to yeast RNA for purified 24-h samples (red) relative to unpurified (black) samples for two biological replicates. The black line represents range. (*B*) Comparisons of human transcript abundance between samples. A heatmap is plotted comparing the abundance of human transcripts in each purified sample, as well as the unpurified (UP) sample. Values in each box are Spearman correlations. (*C*) Comparisons of fly mRNA abundances between samples. As in *B*, except for transcripts encoded in the *Drosophila* genome. (*D*) Ratios of fly to human reads through the time course. The fraction of reads mapping to the human (blue) or *Drosophila* (purple) genome is plotted for each time point. (*E*) The behavior of individual *Drosophila* genes. Plotted is relative abundance for each of the top 10 most highly expressed genes (dashed lines) and the sum of reads mapping to these genes (in red). (*F*) Distribution of half-lives in human cells. The histogram shows the distribution of half-lives in human cells for 12,890 genes. (*G*) Comparison of half-lives between biological replicates. A scatterplot comparing human transcript half-lives for two biological replicates. The red dashed line represents the *x* = *y* line. (*H*) Half-lives calculated using only three time points. A scatterplot comparing half-lives calculated by using all time points or only three (1, 8, and 24 h). The red dashed line represents the *x* = *y* line. See also Supplemental Figure S1.

Because of the variability inherent in using a handful of in vitro transcribed spike-ins, we initially opted to use RNA from two organisms that differed from the species being interrogated. For example, in experiments with HEK293 cells, we used 4SU-labeled RNA from *Drosophila* S2 cells (for normalization) and unlabeled *S. cerevisiae* RNA (to determine the enrichment obtained during the purification). The *Drosophila* and *S. cerevisiae* genomes differ sufficiently enough from the human one that even short reads can be unambiguously assigned (Supplemental Fig. S1A). We routinely observed 100-fold enrichment of 4SU-labeled *Drosophila* RNA compared to unlabeled yeast RNA, indicating that <1% of the signal in the selected population was due to nonspecific background (Fig. 2A). Subsequent analysis indicated that the *Drosophila* S2 RNA was less labeled than the HEK293 RNA (Supplemental Fig. S1B,C), and so these enrichment values likely represent a lower bound.

As incubation with 4SU increased, samples showed reduced similarity in the abundance of human mRNAs (Fig. 2B). Consistent with most transcripts having reached equilibrium by the last time point, the unpurified sample was most similar to the 24 h one and least similar to the 1-h sample (*r*_s_ = 0.98 and *r*_s_ = 0.84, respectively). In contrast, reads mapping to the *Drosophila* genome were unaffected by different harvesting times (Fig. 2C; *r*_s_ = 0.92 to 0.97).

Longer 4SU-incubation times also resulted in a higher fraction of reads mapping to the human genome and a lower fraction to the *Drosophila* genome (Fig. 2D). Although the behavior of individual transcripts from the *Drosophila* spike-in varied, the sum of all reads mapping to the *Drosophila* genome was resistant to outliers (Fig. 2E). We normalized the human-mapping reads by the sum of *Drosophila*-mapping reads, fit a bounded-growth equation to the data, and corrected for a 24-h doubling time. Unlike other approaches (Dölken et al. 2008; Neymotin et al. 2014), we did not rely upon the unselected sample for calculating half-lives, and thus our calculations were unaffected by differential selection biases (such as that reported for longer transcripts [Duffy et al. 2015]).

We calculated half-lives for 12,890 genes in HEK293 cells (Fig. 2F). There was wide variation in transcript stability in these cells, with the most unstable transcript (ID1) having a half-life <15 min, and others having half-lives longer than the cell cycle. For these long-lived transcripts, cell growth and dilution make a major contribution to their dynamics. The half-lives were similar to those we generated with the transcription inhibiters actinomycin D and α-amanitin (Supplemental Fig. S1E,F; *r*_s_ = 0.65 and 0.62, respectively) and with those previously reported (*r*_s_ = 0.62 [Tani et al. 2012]). The half-lives generated with metabolic labeling were generally consistent between biological replicates in their rank order, but less so in magnitude (Fig. 2G; *r*_s_ = 0.61 vs. *r*_p_ = 0.52): in particular, the longest-lived genes varied the most in magnitude between replicates.

In these experiments, we had included six time points in addition to steady-state measurements. However, we hypothesized that not all of these time points would be necessary to determine stabilities, and using fewer samples could reduce the associated library preparation and sequencing costs. We repeated our analysis, but this time omitting individual time points. With the exception of the 24 h time point, the calculated half-lives were robust to the omission of a single time point (Supplemental Fig. S1F; *r*_s_ = 0.95 to 1 vs. *r*_s_ = 0.89). The 24-h time point is likely critical for calculating half-lives, especially for more stable transcripts, because it represents near-equilibrium measurements. Surprisingly, using only three time points (1, 8, and 24 h) gave similar measurements as with all the time points (Fig. 2H; *r*_s_ = 0.94). Although calculations become more robust with more time points, these three represent the minimum requirement for calculating half-lives in human cells using the approach-to-equilibrium method.

### Internal short-lived RNA species can be used to determine mRNA stabilities

In the process of obtaining these half-life measurements, we generated flawed data sets that resulted from insufficient spike-in reads or inconsistent labeling bias, each of which gave a characteristic signature (Supplemental Fig. S2A,B). Such data sets would typically be excluded from downstream analysis, but we wondered if they could be rescued with alternative normalization methods. We hypothesized that because unstable endogenous RNA species quickly reach equilibrium, they could perform a role similar to that of the labeled spike-in RNA.

**FIGURE 3.**
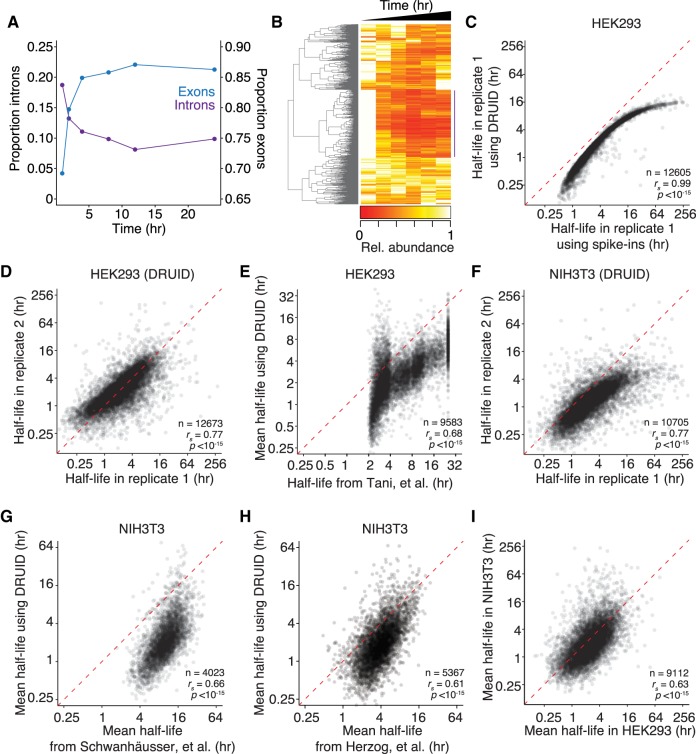
DRUID uses intron measurements to calculate transcript half-lives. (*A*) Behavior of reads mapping to introns during the 4SU-labeling time course. The fraction of reads mapping to exons (blue) or introns (purple) is plotted for each time point in replicate 1. (*B*) Dynamics of intron abundance. Individual introns expressed in HEK293 cells were clustered by their behavior over the 4SU time course in replicate 1. Those introns showing the expected decrease (marked by the purple line) were used in downstream analyses. (*C*) Comparison of half-lives calculated using exogenous spike-ins or DRUID. A scatterplot comparing half-lives in HEK293 cells calculated with exogenous spike-ins or with introns (DRUID). The red dashed line represents the *x* = *y* line. (*D*) Reproducibility of human transcript half-lives calculated using DRUID. A scatterplot comparing half-lives determined using DRUID for two biological replicates. The red dashed line represents the *x* = *y* line. (*E*) Reproducibility of transcript half-lives in NIH3T3 cells calculated using DRUID, otherwise as in *D*. (*F*) Comparison of HEK293 and NIH3T3 transcript half-lives. A scatterplot comparing mean half-lives in HEK293 and NIH3T3 cells. (*G*) Comparison of DRUID-calculated half-lives with published half-lives. A scatterplot comparing the half-lives in NIH3T3 cells calculated with DRUID with those from Schwanhäusser et al. (2011), otherwise as in *D*. (*H*) Comparison of DRUID-calculated half-lives with published half-lives. A scatterplot comparing the half-lives in NIH3T3 cells calculated with DRUID with those from Herzog et al. (2017), otherwise as in *D*. (*I*) Comparison of human and mouse mRNA stabilities. A scatterplot comparing the half-lives in HEK293 cells with those in NIH3T3 cells, otherwise as in *D*.

Consistent with previous observations (Gaidatzis et al. 2015), we noted that introns were abundant in our libraries, especially at the earliest time points, where they made up 19% of the total reads (Fig. 3A). As with the *Drosophila* spike-ins, the overall proportion of reads mapping to introns exhibited a time-dependent decrease (Fig. 3A), and the relative abundance of individual introns did not show a large time-dependent decrease in similarity (Supplemental Fig. S2C; *r*_s_ = 0.90–0.95), indicating that equilibrium levels were generally reached before the first time point. We thus calculated half-lives instead normalizing to intron reads and found significant similarity between replicates (Supplemental Fig. S2D; *r*_s_ = 0.77).

However, we noted that (i) these half-lives were shorter than those determined with the exogenous spike-ins and (ii) not all introns had the same dynamics (Fig. 3B; Supplemental Fig. S2E). We reasoned that improper annotation might affect our calculations, and so we clustered introns based on their behavior over the time-course. We then manually chose the set with a time-dependent decrease in abundance, as would be expected for unstable RNA species. In our first replicate, this cluster contained 1045 introns; another 1564 introns were expressed at sufficient levels, but did not show evidence of being highly unstable. These unexpectedly long-lived introns were likely included in the eventual mature transcript, either due to intron retention or alternative splicing (Braunschweig et al. 2014), or were misannotated.

We next used the sum of all reads mapping to the well-behaving introns (Supplemental Table S1) and calculated half-lives for 12,673 genes in a pipeline we termed “DRUID” (for determination of rates using intron dynamics). Unlike with exogenous spike-ins, DRUID does not require correction for cellular growth. These half-lives again correlated with those obtained by normalizing to exogenous spike-ins (Fig. 3C; *r*_s_ = 0.99), and now they were only slightly shorter, indicating that improper intron annotation had impacted the absolute magnitude of our original, intron-based calculations (Supplemental Fig. S2E,F).

DRUID yielded half-lives significantly more similar between replicates than those we obtained earlier with exogenous spike-ins (*r*_s_ = 0.77 vs. *r*_s_ = 0.61; Fisher's R-to-z transformation, *P* < 10^–50^). Moreover, the skew that we had observed between replicates for long-lived transcripts was not apparent when we used DRUID (Fig. 3D cf. Fig. 2G), indicating that DRUID gives reproducible rank order and magnitudes for mRNA half-lives (*r*_s_ = 0.77 vs. *r*_p_ = 0.74). Intron normalization likely captures in vivo experimental variation better than the exogenous spike-ins and is thus better equipped to normalize for these differences. When we generated half-lives using only three time points (1, 8, and 24 h), DRUID gave similar results (Supplemental Fig. S2G; *r*_s_ = 0.92) and was consistent between replicates (*r*_s_ = 0.73).

We next used a previously published set of human cell line half-lives (Tani et al. 2012) to benchmark the four different sets of half-lives we had determined: namely, transcriptional inhibition with actinomycin D or α-amanitin, metabolic labeling with normalization to exogenous standards, and metabolic labeling with normalization to endogenous standards (i.e., DRUID). Of the four sets, half-lives determined by metabolic labeling and normalization to exogenous standards performed the worst when compared to the benchmarking data set (Supplemental Fig. S3A; *r*_s_ = 0.46; Fisher's R-to-z transformation, *P* < 10^–16^), and half-lives determined by either transcriptional inhibition method were significantly more correlated (Supplemental Fig. S3B,C; *r*_s_ = 0.56–0.58; Fisher's R-to-z transformation, *P* < 10^–16^). However, despite being derived from the same raw data sets as those for exogenous standards, DRUID-calculated half-lives outperformed the other three sets and were significantly more correlated with the benchmarking data set (Fig. 3E; *r*_s_ = 0.68; Fisher's R-to-z transformation, *P* < 10^–26^). Similarly, DRUID-calculated half-lives performed significantly better than exogenous-standard–derived half-lives when each was compared with half-lives determined by transcription inhibition (Supplemental Fig. S3D,E; Fisher's R-to-z transformation, *P* < 10^–100^). Thus, we conclude that DRUID represents a powerful computational method for calculating half-lives.

### Orthologous mouse and human genes have similar mRNA half-lives

To further confirm the applicability of our method, we used DRUID to calculate mRNA half-lives in NIH3T3 cells again using a restricted intron set (Supplemental Table S2). We obtained measurements for 10,705 genes with high similarity between replicates (Fig. 3E; *r*_s_ = 0.77). As with our HEK293 experiments, DRUID performed better than using exogenous spike-ins for normalization (Supplemental Fig. S2E; Fisher's R-to-z transformation, *P* < 10^–30^). These values were similar to those previously calculated (Supplemental Fig. S2F–H; *r*_s_ = 0.66 [Schwanhäusser et al. 2011]; *r*_s_ = 0.61 [Herzog et al. 2017]) although using our method we were able to determine half-lives for a larger number of genes (<6000 vs. 10,705).

We next compared mRNA half-lives and equilibrium levels of orthologous human and mouse genes (Fig. 3F; Supplemental Fig. S2G). Surprisingly, given that these two cell lines are from different organisms and derived from different cell types, we found a high correlation between RNA abundance and half-lives between mouse and human orthologs (*r*_s_ = 0.57 and 0.63, respectively). Thus, although some transcripts, such as *MBNL3*, display striking differences in stability between HEK293 and NIH3T3 cells (26 h vs*.* 36 min, respectively), many conserved transcripts are degraded at similar rates.

### DRUID can retroactively rescue data sets

**FIGURE 4.**
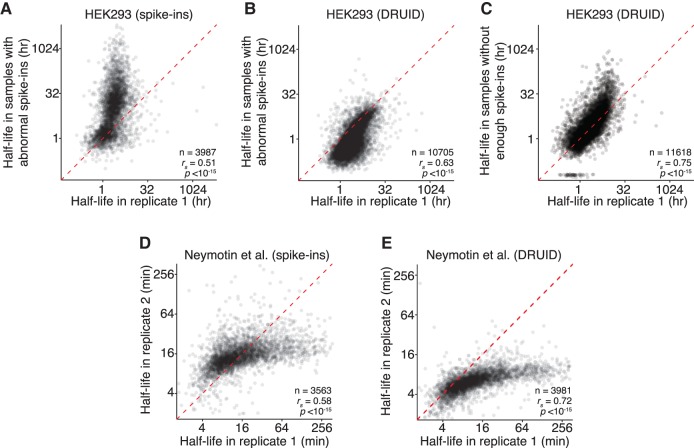
DRUID can rescue recalcitrant data sets. (*A*) Effect of poorly behaving data sets on transcript half-lives calculated with exogenous spike-ins. A scatterplot comparing the half-lives in HEK293 cells calculated in one biological replicate and a recalcitrant data set with poorly behaving spike-ins. The red dashed line represents the *x* = *y* line. (*B*) Comparison of transcript half-lives calculated with DRUID. As in *A*, except half-lives were calculated using DRUID. (*C*) Comparison of half-lives calculated with DRUID. As in *B*, except for a second, recalcitrant data set, whose spike-ins behaved so poorly that no half-lives could be calculated using exogenous spike-ins. (*D*) Comparison of yeast half-lives calculated using exogenous spike-ins, otherwise as in *A*. (*E*) Comparisons of yeast half-lives calculated using DRUID, otherwise as in *A*. See also Supplemental Figure S2.

Having established that DRUID can be used on high quality data sets, we finally asked whether this normalization method could rescue previously unusable data sets. We focused on two data sets: one with too few spike-in reads and the other with abnormal spike-in behavior (Supplemental Fig. S2A,B). In both cases, this behavior of the exogenous spike-ins resulted in normalized dynamics for endogenous genes that only poorly fit to the bounded growth equation: For the first data set, we were unable to calculate any half-lives; for the second, we obtained half-lives for only 3987 genes. Although measurements from the second set did correlate with other replicates (Fig. 4A, *r*_s_ = 0.51), we observed a strong skew for long-lived transcripts.

Strikingly, for both data sets, intron normalization was able to overcome both types of technical difficulties, and we generated half-lives for over 10,000 genes. (As noted earlier, the number of genes for which half-lives can be determined is affected by normalization strategies.) These half-lives correlated well with our other data sets (Fig. 4B,C; *r*_s_ = 0.63–0.75). Importantly, we no longer observed the difference in half-life magnitude for stable transcripts that we saw with exogenous normalization (Fig. 4A vs. Fig. 4B). Thus, DRUID can be used for otherwise recalcitrant data sets.

One potential drawback of intron normalization is its applicability to organisms with few introns, such as *S. cerevisiae*. We thus applied DRUID to published data sets from budding yeast (Neymotin et al. 2014). When we calculated half-lives using the three exogenous spike-in transcripts originally included in this experiment, RNA half-lives were correlated (Fig. 4D; *r*_s_ = 0.58). However, DRUID generated half-lives that were significantly more correlated (Fig. 4E; *r*_s_ = 0.72; Fisher's R-to-z transformation, *P* < 10^–70^) and for a larger number of genes (3563 vs. 3981). We note that, irrespective of the computational scheme, the magnitude of half-lives differed between these two experiments, suggesting that there may be additional technical differences, such as labeling bias, that cannot be completely accounted for by DRUID. Thus, these results demonstrate that normalizing to introns can be used on data sets not originally intended for DRUID. Furthermore, DRUID is a robust and widely applicable normalization method, appropriate even for organisms with few introns.

## DISCUSSION

Despite known and important issues in transcriptional shut-off approaches, RNA polymerase inhibitors remain in common use for determining transcript stability. To enable wider adoption of approach-to-equilibrium metabolic labeling strategies, we developed DRUID, a computational method that robustly calculates mRNA half-lives on a transcriptome-wide scale. Although we initially envisioned using exogenous spike-ins for a normalization approach, we were surprised that this framework was surpassed by intron normalization. Despite using oligo(dT) selection to generate our libraries, we found that introns were abundant in our data sets; these reads are possibly derived from processing intermediates and are consistent with previous observations (Gaidatzis et al. 2015). We found that intron-based normalization was effective for all data sets we examined, irrespective of the organism examined, even for data sets that were otherwise recalcitrant. Our computational pipeline is publicly available to enable wider use of the approach-to-equilibrium strategy (see Materials and Methods).

Due to the wide use of 4SU-labeling followed by biotinylation (Rabani et al. 2011, 2014; Neymotin et al. 2014), here we have focused on data sets derived from approach-to-equilibrium 4SU-labeling experiments. However, DRUID can be used with data sets from any approach-to-equilibrium labeling experiments and is agnostic to the specific biochemical approach to purify labeled RNA. There are two main requirements for DRUID, as there are with all approach-to-equilibrium strategies (Greenberg 1972; Ross 1995). First, the labeling reagent, such as 4SU, must be readily taken up by the cell and incorporated into newly synthesized transcripts at concentrations that do not have negative physiological effects. Second, an underlying assumption of metabolic labeling and DRUID is that the system is at steady state. Thus, in its current form, DRUID cannot be used to investigate scenarios where rates of synthesis and decay change throughout the experiment. Of course, biological processes, such as differentiation, are frequently defined by changes in both mRNA transcription and decay, and so an important next step will be to generate experimental and computational methods that can monitor dynamic systems while remaining accessible to the broader community.

Given the success of DRUID for calculating half-lives, is there any utility for including exogenous spike-ins? Although in principle they are not required, in practice we still routinely include them in our experiments for two reasons. First, our exogenous spike-in strategy allows us to calculate the enrichment of labeled RNA in each data set, thus confirming that the purification has worked as expected. Second, and more importantly, the exogenous spike-ins provide an independent normalization scheme and thus a useful quality control. Comparing between normalization schemes greatly increases confidence in the calculated half-lives and is particularly important when new cell types or systems are being used.

## MATERIALS AND METHODS

### Cell lines and strains

Human HEK293 epithelial cells (ATCC CRL1573) were cultured in Eagle's minimum essential medium (EMEM) supplemented with 10% fetal bovine serum (FBS) and 1% penicillin–streptomycin solution. Murine NIH3T3 fibroblasts (ATCC CRL1658) were cultured in Dulbecco's modified Eagle's medium (DMEM) supplemented with 10% donor calf serum (DCS) and 1% penicillin–streptomycin solution. Both mammalian cell lines were cultured at 37°C in a humidified incubator with 5% CO_2_. *Drosophila melanogaster* Schneider 2 (S2) cells (Thermo Fisher Scientific R69007) were cultured in ExpressFive SFM media (Thermo Fisher Scientific), supplemented with 10% heat-inactivated FBS and 20 mM L-Glutamine, at 28°C.

*S. cerevisiae* USY006 was grown in YPD liquid or plates at 30°C. RNA was isolated using the standard hot phenol method (Rissland and Norbury 2009). Synchronized populations of L1 *C. elegans* were grown on NGM plates for 60 h until adult staged. Worms were washed off of plates with PBS buffer and resuspended in ultrapure water. RNA was extracted using TRI reagent (Molecular Research Center), according to manufacturer's instructions.

### Metabolic labeling

For metabolic labeling experiments, cells were treated with 100 µM 4SU and harvested after 1, 2, 4, 8, 12, and 24 h. S2 cells treated with 100 µM 4SU for 24 h were used for the generation of labeled spike-ins. When harvesting adherent cells, cells were dislodged with PBS and subjected to two PBS washes. S2 cells were pelleted and subjected to two PBS washes. RNA was extracted using TRI reagent (Molecular Research Center), according to manufacturer's instructions.

### Transcription shut-off experiments

HEK293 cells were treated with either 5 µg/mL actinomycin D or 50 µg/mL α-amanitin for 0, 1, 2, 4, 8, 12, and 24 h and were harvested as described above.

### In vitro biotinylation and biotin–streptavidin pull down

A 1 mg/mL solution of HPDP-biotin (Thermo Fisher Scientific) in dimethylformamide was incubated at 37°C for 30 min. Of note, 40–100 µg of RNA was combined with 20% w/w unlabeled yeast RNA and 20% w/w 4SU labeled fly RNA (human RNA for fly samples) and 120 µL of 2.5× citrate buffer (25 mM citrate pH 4.5, 2.5 mM EDTA) in a total volume of 240 µL. A total of 60 µL of HPDP–biotin solution was added, and the RNA was incubated for 2 h at 37°C, covered and shaken at 300 rpm. RNA was then phenol–chloroform extracted and ethanol precipitated with 2 µL of glycoblue (Life Technologies). The RNA pellet was resuspended in 200 µL of 1× wash buffer (10 mM Tris–Cl pH 7.4, 50 mM NaCl, 1 mM EDTA).

A total of 50 µL of MACS microbeads (Miltenyi Biotec) were incubated with 48 µL of 1× wash buffer and 2 µL of yeast tRNA for 20 min at room temperature with rotation. MACS microcolumns (Miltenyi Biotec) were washed with 100 µL of nucleic acid equilibration buffer (Miltenyi Biotec) and then five times with 100 µL of 1× wash buffer. Beads were applied to the column in 100 µL aliquots and washed five times with 100 µL of 1× wash buffer. Columns were demagnetized and beads eluted with two 100 µL washes with 1× wash buffer, and columns were remagnetized. The 200 µL bead solution was combined with RNA sample and rotated at room temperature for 20 min. The sample was then applied to the column in 100 µL aliquots. Columns were washed three times with 400 µL of wash 1 buffer (10 mM Tris–Cl pH 7.4, 6 M urea, 10 mM EDTA) prewarmed to 65°C and then three times with 400 µL wash 2 buffer (10 mM Tris-Cl pH 7.4, 1 M NaCl, 10 mM EDTA). RNA bound to the column was eluted with five washes of 1× wash buffer with 0.1 M dithiothreitol, and then ethanol precipitated with 2 µL of glycoblue.

### RNA sequencing

Sequencing libraries were prepared using the TruSeq Stranded mRNA Sample Preparation Kit (Illumina), according to manufacturer's instruction manual (Rev. E), and sequenced at The Centre for Applied Genomics (SickKids).

### Computational analysis: read mapping

Libraries were pooled and sequenced on an Illumina HiSeq 2500 to give 50 bp single-end reads. RTA v1.18.54 or later was used for base calling and quality scores, bcl2fastq2 v2.17 or later was used to demultiplex samples and to convert reads to fastq format. Library quality was assessed using FastQC v0.11.5 (http://www.bioinformatics.babraham.ac.uk/projects/fastqc/). Reads were trimmed and clipped for Illumina adaptors using Trimmomatic v0.36 (Bolger et al. 2014) with the following options: LEADING:3 TRAILING:3 SLIDINGWINDOW:4:15 MINLEN:36. Reads were aligned to merged reference genomes (hg38 + sacCer3; hg38 + dm6 + sacCer3; hg38 + dm6 + ce10; mm10 + dm6 + sacCer3) obtained using the UCSC Table Browser (Karolchik et al. 2004; Rosenbloom et al. 2015) and kentUtils v302 using STAR version 2.5.2a_modified (Dobin et al. 2013). STAR was invoked with default settings aside from outFilterMultimapNmax 10, outFilterMismatchNoverLmax 0.05, outFilterScoreMinOverLread 0.75, outFilterMatchNminOverLread 0.85, alignIntronMax 1, and outFilterIntronMotifs RemoveNoncanonical.

Mapped reads were quantified using two different methods. First, an in-house R script (v3.2.3) was used to select the longest transcript for every gene, using the GenomicFeatures (Lawrence et al. 2013), rtracklayer (Lawrence et al. 2009), and plyr packages as well as packages from Bioconductor (Gentleman et al. 2004; Huber et al. 2015). Any introns that overlapped with an exon in any other isoform were removed. HTSeq 0.6.1p2 (Anders et al. 2015) was used to count reads mapping to introns and exons. In addition to HTSeq, an in-house intersection method was used to calculate intron coverage, based on tools provided by the BEDTools suite v2.26.0 (Quinlan and Hall 2010). Briefly, coverage was determined at the nucleotide level and subsequently averaged at the intron level, providing finer quantitation than a simple count-based approach. This quantitation method is available in the DRUID GitHub repository and is the recommended method for downstream analysis with DRUID.

### Computational analysis: half-life determination

All downstream analyses were performed using in-house R scripts utilizing the follow libraries: scales, plyr, gplots, Hmisc, and limma (Ritchie et al. 2015). Read counts were first filtered to require that each gene had a minimum of one mapped read in all time points with five or more mapped reads in at least one time point. Transcriptomic reads were normalized to spike-ins.

For transcription inhibition experiments, half-lives were determined by fitting an exponential decay model to normalized data using nonlinear least squares. Exponential decay is described by *N*(*t*) = *N*_0_e^−λt^, where *N*(*t*) is the amount of transcript remaining at time *t*, *N*_0_ is the amount of a transcript at steady state, and λ is the transcript-specific decay constant. The transcript-specific half-life (hl) can then be obtained with the simple equation, hl = ln(2)/λ.

For 4SU time courses, a bounded growth equation was fit using weighted nonlinear least squares. Using the above notation, the bounded growth equation can be written as *N*(*t*) = *N*_0_(1−e^−(λ+γ)*t*^), where the additional term, γ, is the dilution due to growth and can be calculated using the doubling time (δ) of the model under study by the equation γ = ln(2)/δ.

In DRUID, introns were used for normalization. In order to quantify intron abundance, introns were filtered such that the mean coverage spanning the intron was 0.5 reads and clustered based on their time-dependent expression profiles using *k*-means clustering with four clusters. The cluster exhibiting behavior closest to the expected nonincreasing time-dependent abundance was chosen manually. Exon-mapping reads were then normalized to the sum of all reads mapping to the well-behaved intron set. As before, to calculate half-lives, a bounded growth equation was fit using weighted nonlinear least squares. DRUID is available on GitHub: https://github.com/risslandlab/DRUID. A list of human–mouse orthologs was downloaded from Mouse Genome Informatics (http://www.informatics.jax.org/).

For half-lives derived from other studies, we used the published half-lives (Schwanhäusser et al. 2011; Tani et al. 2012; Herzog et al. 2017) or, in the case of Neymotin et al. (2014), calculated the half-lives using either exogenous normalization or DRUID, as described above.

## DATA DEPOSITION

Data generated in this study are available from the GEO, accession number GSE99517.

## SUPPLEMENTAL MATERIAL

Supplemental material is available for this article.

## Supplementary Material

Supplemental Material
