# Supplementary material for: DRUID: a pipeline for transcriptome-wide measurements of mRNA stability
Source: RNA. 2018 May;24(5):623–32. doi: 10.1261/rna.062877.117 (PMC5900561; doi:10.1261/rna.062877.117)
Supplement: Supplemental Material [file supp_24_5_623__index.html]

DRUID: a pipeline for transcriptome-wide measurements of mRNA stability — Supplemental Material 

# DRUID: a pipeline for transcriptome-wide measurements of mRNA stability

## Supplemental Material

- Supplemental\_Figure\_S1.pdf
- Supplemental\_Figure\_S2.pdf
- Supplemental\_Figure\_S3.pdf
- Supplemental\_Table\_S1.csv
- Supplemental\_Table\_S2.csv
- Supplemental\_Table\_S3.csv
