# Supplementary material for: DRUID: a pipeline for transcriptome-wide measurements of mRNA stability
Source: RNA. 2018 May;24(5):623–32. doi: 10.1261/rna.062877.117 (PMC5900561; doi:10.1261/rna.062877.117)
Supplement: Supplemental Material [file supp_062877.117_Supplemental_Figure_S1.pdf]

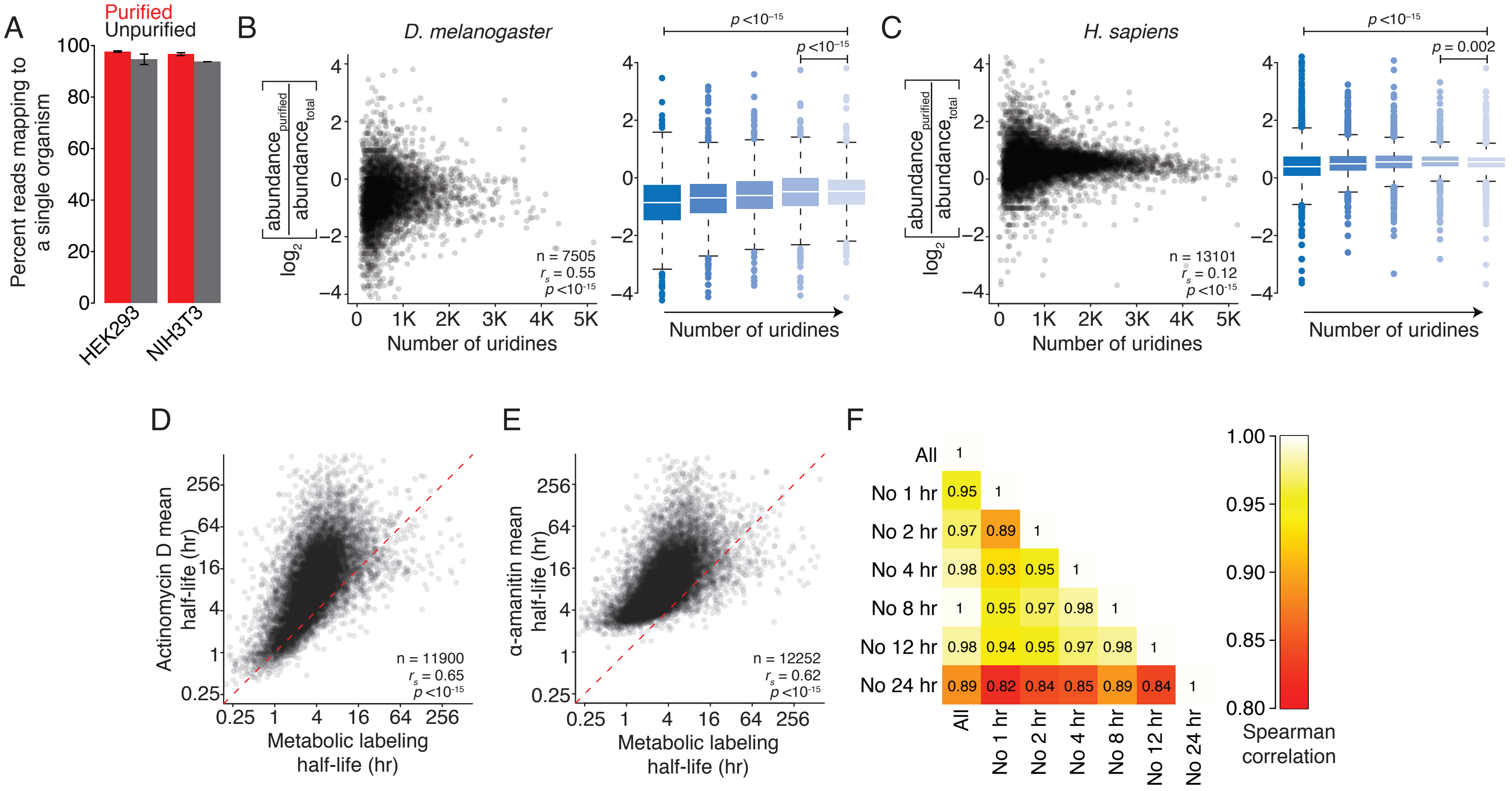

Supplemental Figure S1. The behavior of approach-to-equilibrium half-lives using exogenous spike-ins.(A) The fraction of reads mapping unambiguously to the different genomes. Shown is the fraction of reads that map uniquely to a combined human–yeast–fly genome (left) or a combined mouse–yeast–fly genome (right) for unpurified (in grey) and purified (in red) samples. (B) The effect of the number of uridines on selection efficiency for Drosophila mRNAs. Left, a scatterplot comparing the number of uridines with their relative enrichment in the purified 24 hr sample. Right, box-and-whisker representation of the scatterplot. Box represents 25th to 75th percentile; whiskers, the range, excluding outliers. (C) The effect of the number of uridines on selection efficiency for human mRNAs, otherwise as in (B). (D) Comparison of 4SU-determined half-lives with those determined by actinomycin D. Plotted is a scatterplot comparing half-lives calculated using metabolic labeling and the mean half-lives (of 2 biological replicates) calculated with actinomycin D. The red dashed line represents the x = y line. (E) Comparison of 4SU-determined half-lives with those determined by α-amanitin, otherwise as in (D). (F) Comparison of half-lives determined with individual time-points omitted. Shown is a heat map comparing half-lives calculated with the indicated time points omitted. The values in each box correspond to the Spearman correlation; n = 10,680.
