# Supplementary material for: DRUID: a pipeline for transcriptome-wide measurements of mRNA stability
Source: RNA. 2018 May;24(5):623–32. doi: 10.1261/rna.062877.117 (PMC5900561; doi:10.1261/rna.062877.117)
Supplement: Supplemental Material [file supp_062877.117_Supplemental_Figure_S2.pdf]

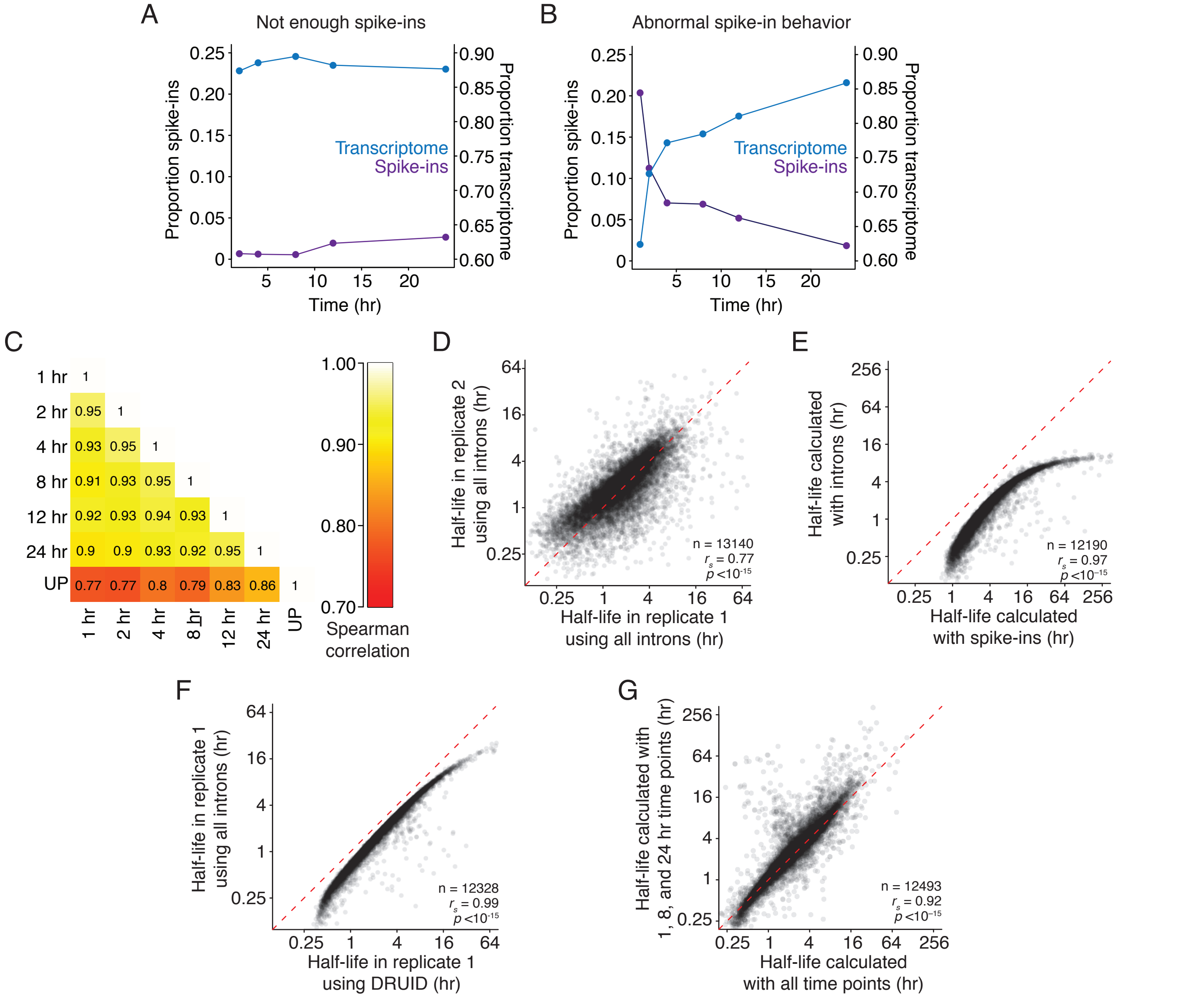

Supplemental Figure S2. The behavior of approach-to-equilibrium half-lives using DRUID.
(A) Ratios of fly to human reads through the time course for recalcitrant dataset 1. The fraction of reads mapping to the human (in blue) or Drosophila (in purple) genome is plotted for each time point. Here, insufficient levels of exogenous spike-ins meant that half-lives could not be calculated. (B) Ratios of fly to human reads through the time course for recalcitrant dataset 2, otherwise as in (A). Here, the exogenous spike-ins exhibited abnormal behavior, and calculated half-lives were not reproducible. (C) Comparisons of human intron abundance between samples. A heatmap is plotted comparing the calculated human intron abundance in each purified sample, as well as the unpurified (UP) sample. Values in each box are Spearman correlations; n = 11,460. (D) mRNA half-lives calculated by intron normalization. A scatterplot comparing the mRNA half-lives in two replicates using intron-normalization. The red dashed line represents the x = y line. (E) Comparison of half-lives determined by exogenous spike-in and intron normalization, otherwise as in (D). (F) The effect of restricting introns used in calculated half-lives. A scatterplot comparing mRNAs half-lives using all introns or only the well-behaved set. Otherwise as in (D). (G) mRNA half-lives calculated using only three time points. A scatterplot comparing DRUID mRNA half-lives using all of the time points or only three (1, 8, and 24 hr),otherwise as in (D).
