# Supplementary material for: DRUID: a pipeline for transcriptome-wide measurements of mRNA stability
Source: RNA. 2018 May;24(5):623–32. doi: 10.1261/rna.062877.117 (PMC5900561; doi:10.1261/rna.062877.117)
Supplement: Supplemental Material [file supp_062877.117_Supplemental_Figure_S3.pdf]

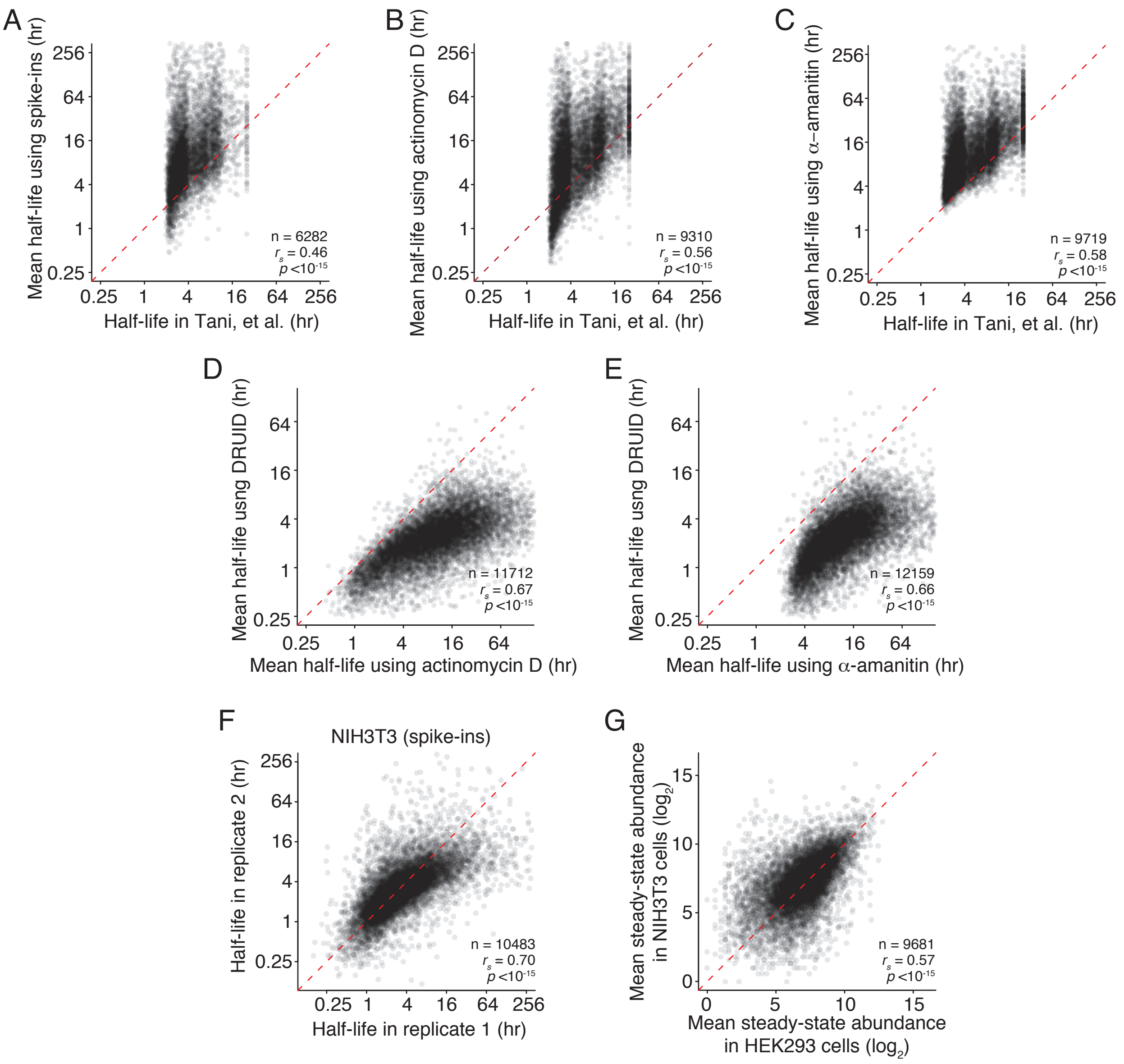


**Supplemental Figure 3. Comparison of mRNA half-lives determined by different methods.** (A) Comparison of half-lives determined in Tani, et al. and using metabolic labeling with normalization to exogenous spike-ins. The red dashed line represents the x = y line. (B) As in (A), except comparing half-lives determined in Tani, et al. and with actinomycin D. (C) As in (A), except comparing half-lives determined in Tani, et al. and with α-amanitin. (D) As in (A), except comparing half-lives determined with DRUID and actinomycin D. (E) As in (A), except comparing half-lives determined with DRUID and α-amanitin. (F) Comparison of mRNA half-lives in NIH3T3 cells between two biological replicates, otherwise as in (D). The red dashed line represents the x = y line. (G) Comparison of HEK293 and NIH3T3 mRNA steady-state abundance. A scatterplot comparing mean mRNA FPKM values in HEK293 and NIH3T3 cells, otherwise as in (A).
